# Duodromic atrioventricular reentry tachycardia: a case report of a rare adenosine insensitive supraventricular tachycardia

**DOI:** 10.1093/ehjcr/ytae698

**Published:** 2024-12-30

**Authors:** Sofia Jacinto, Margarida Figueiredo, Inês Almeida, Bruno Valente, Mário Martins Oliveira

**Affiliations:** Arrhythmology, Pacing and Electrophysiology Unit, Cardiology Service, Santa Marta Hospital, Central Lisbon Hospital University Centre, R. de Santa Marta 50, Lisboa 1169-024, Portugal; Arrhythmology, Pacing and Electrophysiology Unit, Cardiology Service, Santa Marta Hospital, Central Lisbon Hospital University Centre, R. de Santa Marta 50, Lisboa 1169-024, Portugal; Arrhythmology, Pacing and Electrophysiology Unit, Cardiology Service, Santa Marta Hospital, Central Lisbon Hospital University Centre, R. de Santa Marta 50, Lisboa 1169-024, Portugal; Arrhythmology, Pacing and Electrophysiology Unit, Cardiology Service, Santa Marta Hospital, Central Lisbon Hospital University Centre, R. de Santa Marta 50, Lisboa 1169-024, Portugal; Arrhythmology, Pacing and Electrophysiology Unit, Cardiology Service, Santa Marta Hospital, Central Lisbon Hospital University Centre, R. de Santa Marta 50, Lisboa 1169-024, Portugal

**Keywords:** Ablation, Accessory pathways, Atrioventricular reentry tachycardia, Case Report, Supraventricular tachycardia, Ventricular pre-excitation, Wolff–Parkinson–White

## Abstract

**Background:**

Accessory pathways (AP) are associated with an increased risk of atrioventricular reentry tachycardia (AVRT), presenting as a wide QRS tachycardia if the mechanism is antidromic. Rarely, AVRT may not respond to adenosine, suggesting a duodromic mechanism if the patient has multiple APs. Herein, we present a case of a male patient with multiple APs, wide QRS complex tachycardia, and resistance to adenosine.

**Case presentation:**

A 45-year-old man with Wolff–Parkinson–White (WPW) syndrome was referred for AP ablation. He had previously been admitted with persistent palpitations and wide QRS tachycardia, which was resistant to adenosine. Electrophysiologic study revealed both right lateral and left lateral APs. Ablation successfully eliminated conduction through both pathways. Six months later, the patient remained asymptomatic but exhibited recurrence of pre-excitation on electrocardiogram, suggesting the presence of a third AP. A repeat electrophysiology study confirmed a posteroseptal AP, which was successfully ablated. The patient remained free of pre-excitation at follow-up.

**Discussion:**

This case highlights the complexity of the diagnosis and treatment of wide QRS tachycardias in a patient with WPW. In this case, the failure to respond to adenosine was attributed to the use of a second AP as the retrograde limb of the AVRT circuit, a rare phenomenon known as duodromic AVRT. Successful identification and ablation of all APs was crucial in preventing recurrent arrhythmias, and rare mechanisms such as duodromic tachycardia should be considered when standard treatments fail.

**Learning points**

- Patients with multiple accessory pathways (APs) are at higher risk of developing arrhythmias such as atrioventricular reentry tachycardia (AVRT), atrial fibrillation, and ventricular fibrillation.
- Antidromic AVRT may present as a wide QRS tachycardia which typically is responsive to adenosine.
- Failure of adenosine to terminate the tachycardia should raise suspicion of a duodromic mechanism, in which two APs contribute to the circuit (one as an antegrade limb and the other as a retrograde limb).
- Electrophysiologic study plays a vital role in diagnosing and mapping APs, particularly in patients resistant to pharmacological treatment. Electroanatomic mapping systems can accurately localize APs for targeted ablation.

## Introduction

Accessory pathways (AP) result from abnormal embryological development during the differentiation of fibrous tissue that separates the atria and ventricles.^1^ Wolff–Parkinson–White (WPW) syndrome, referring to the presence of a manifest AP with pre-excitation on the electrocardiogram (ECG) in combination with recurrent tachyarrhythmias, has an estimated prevalence of 0.15–0.25% in the general population, and may predispose patients to atrioventricular reentry tachycardia (AVRT), atrial fibrillation (AF), and sudden cardiac death.^2^

Multiple APs are estimated to occur in 3–13% of patients that undergo electrophysiology study (EPS) for the diagnosis of AP.^3^ Patients with multiple APs are at even higher risk for supraventricular tachycardias, with the potential for rapid anterograde AP conduction during AF, which may lead to ventricular fibrillation.^3,4^ Even less commonly, reentry can use two separate APs without participation from the AV node, resulting in a duodromic AVRT.

Herein, we report the case of a male patient with a manifest AP on a routine ECG, who was admitted to the emergency room (ER) with a wide QRS complex tachycardia and no response to antiarrhythmic therapy with adenosine.

## Summary figure

**Figure:**
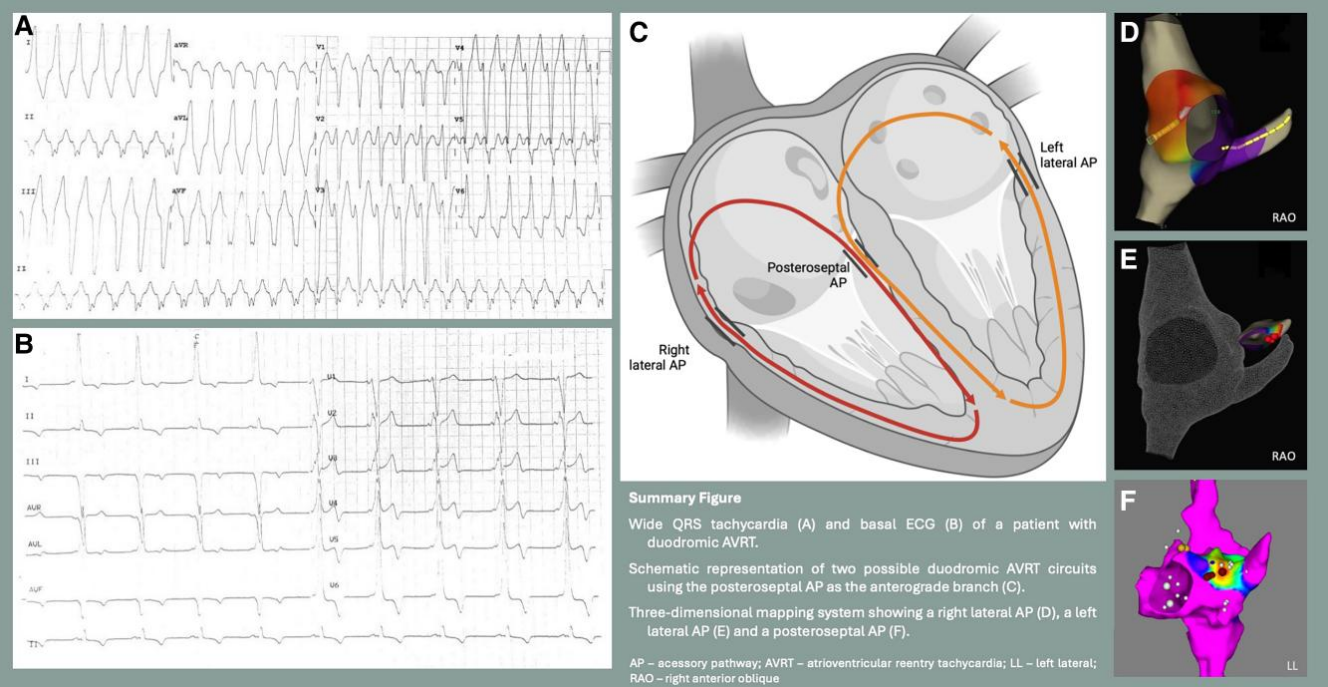


## Case presentation

**Figure 1:**
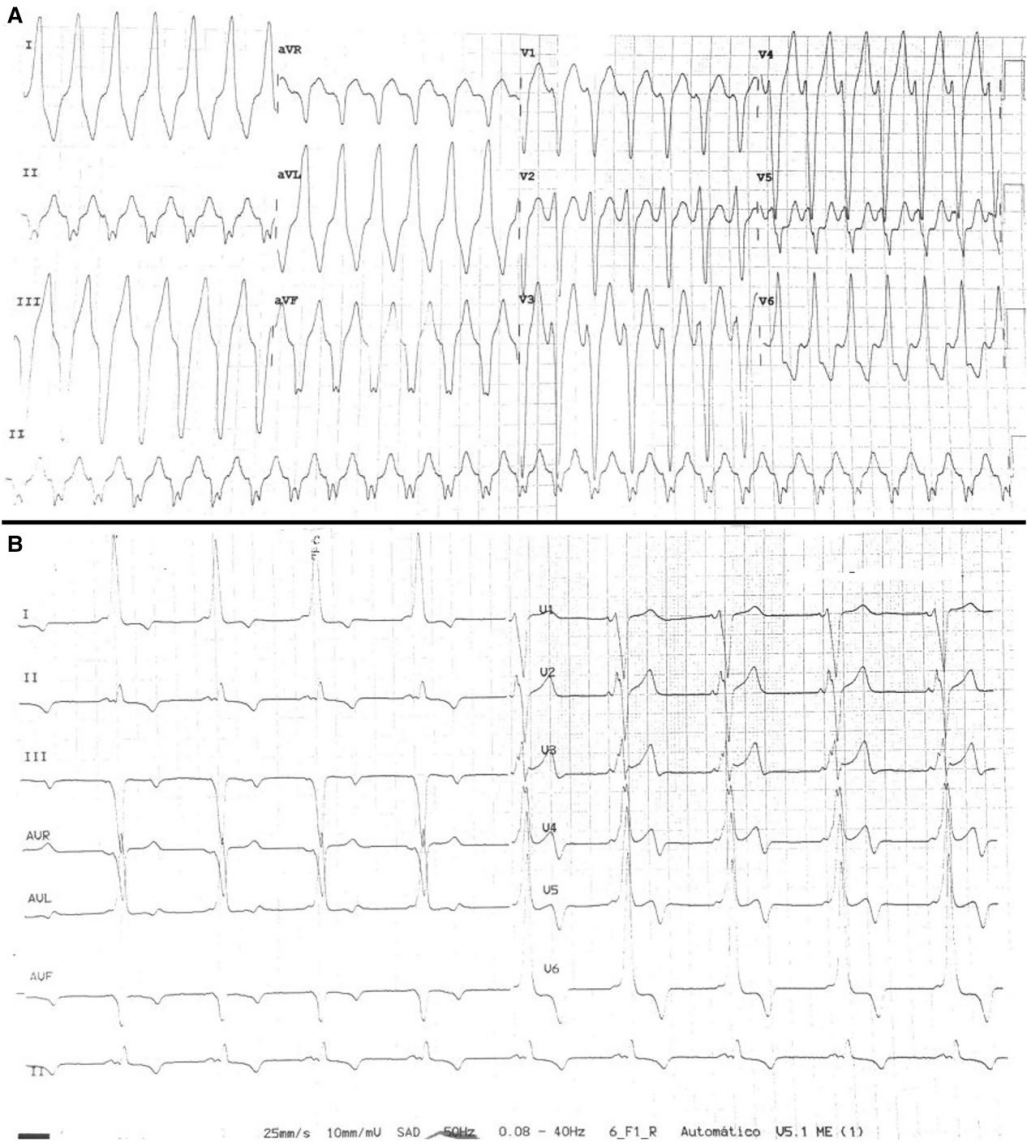
Electrocardiogram at admission in the emergency room showing a regular wide QRS tachycardia (*A*); and after amiodarone administration showing ventricular pre-excitation with a morphology suggestive of a right posterior accessory pathway (*B*).

A 45-year-old man with WPW syndrome, presenting with recurrent palpitations, was referred to a tertiary hospital for ablation of an AP. He had no other relevant medical history and was medicated with bisoprolol 5 mg i.d. He had previously been admitted to the ER for persistent, rapid palpitations and a haemodynamically tolerated regular tachycardia, with a cycle length of 377 ms, wide QRS complexes with a left bundle branch block pattern and superior axis (*Figure 1A*). On physical examination, the patient was oriented, with a blood pressure of 114/78 mmHg, heart rate of 159 b.p.m., peripheral oxygen saturation of 98%, and axillar temperature of 36°C. Cardiac auscultation was rhythmic and tachycardiac, without murmurs, and the remaining physical examination was unremarkable. In the ER, intravenous propranolol (2 mg) was administered first, followed by adenosine boluses (12 mg initially and 18 mg subsequently) with no interruption of the arrhythmia. The arrhythmia later terminated under amiodarone infusion. The basal ECG in sinus rhythm showed ventricular pre-excitation with morphology suggestive of a right posterior AP (*Figure 1B*). Transthoracic echocardiogram revealed preserved biventricular function, without regional wall motion abnormalities or haemodynamically significant valve disease. Laboratory tests were unremarkable, including haemoglobin 15.0 g/dL [normal range (NR): 13.5–17.5 g/dL], white blood cell count 6500/µL (NR: 4000–11 000/µL), platelet count 247 000/µL (NR: 150 000–450 000/µL), sodium 138 mEq/L (NR: 135–145 mEq/L), potassium 4.1 mEq/L (NR: 3.5–5.0 mEq/L), blood urea nitrogen 14 mg/dL (NR: 7–20 mg/dL), creatinine 0.9 mg/dL (NR: 0.6–1.3 mg/dL), total bilirubin 0.8 mg/dL (NR: 0.1–1.2 mg/dL), and TSH 2.0 µIU/mL (NR: 0.4–4.0 µIU/mL).

**Figure 2:**
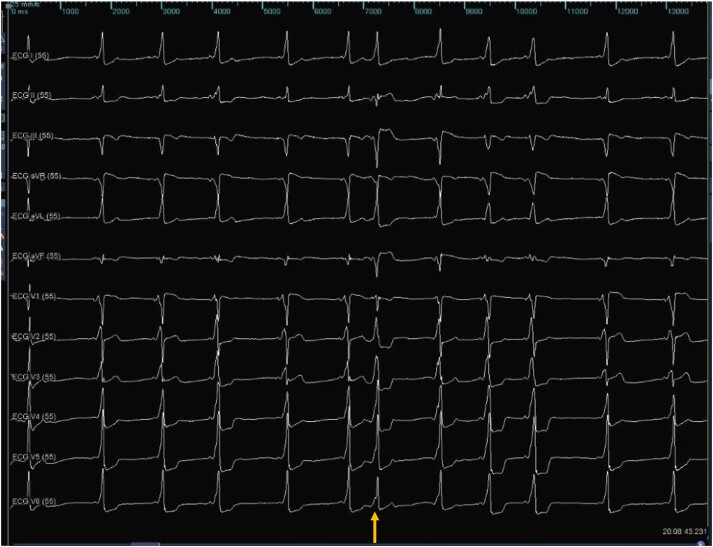
Surface electrocardiogram at the beginning of the electrophysiology study showing anterograde ventricular activation with pre-excitation through a right lateral accessory pathway. A premature atrial contraction (↑) with conduction through a posteroseptal pathway can be suspected with a more negative deflection in aVF.

**Figure 3:**
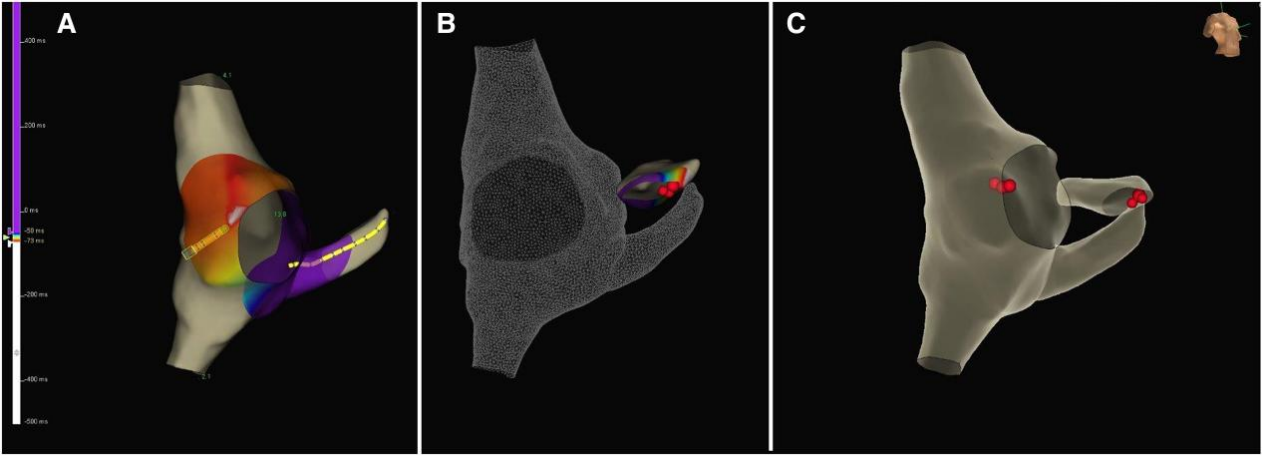
EnSite Precision™ 3D images showing the local activation time of both the right (*A*) and left (*B*) accessory pathways. Radiofrequency ablation was performed in both locations (*C*).

An EPS was performed under local anaesthesia. Surface ECG at the beginning of the study showed anterograde activation with pre-excitation through a right lateral pathway, with a single premature atrial contraction compatible with conduction through a posteroseptal pathway (*Figure 2*). However, intracardiac electrograms during ventricular stimulation showed eccentric, non-decremental ventricular-atrial (VA) conduction, consistent with a left AP. Despite programmed atrial stimulation before and during isoproterenol infusion (0.5 μg/min until a heart rate of 170 b.p.m.), a circus movement tachycardia was not inducible. Intracardiac mapping was performed with a high-density mapping catheter (Advisor HD™ Grid, Abbott) and EnSite Precision™ (Abbott) electroanatomic mapping system (*Figure 3*). A FlexAbility™ (Abbott) irrigated ablation catheter was positioned at the tricuspid annulus, at the site where the shortest atrioventricular (AV) interval was observed. Radiofrequency (RF) was applied (40W, 15 mL irrigation, maximum temperature of 42°C), resulting in the disappearance of AP conduction within the first 5 s (*Figure 3A*). A transseptal puncture was then performed to access and map the left cardiac chambers. Retrograde mapping during ventricular pacing revealed a shorter VA interval, with continuous electrical activity near the 2/1 dipole of the coronary sinus (CS) catheter. Radiofrequency was also applied at this site (40W, 15 mL irrigation, maximum temperature of 42°C), successfully eliminating conduction through this pathway (*Figure 3B*). After a 20-min observation period, there was no recurrence of manifest pre-excitation. Ventricular-atrial dissociation was documented during ventricular stimulation (see Supplementary material online, *F*igure  *S1A*) and atrial pacing resulted in a narrow QRS with absence of pre-excitation (see Supplementary material online, *F*igure  *S1B*). Ablation of both the right lateral and left lateral pathways was considered successful (*Figure 3C*).

**Figure 4:**
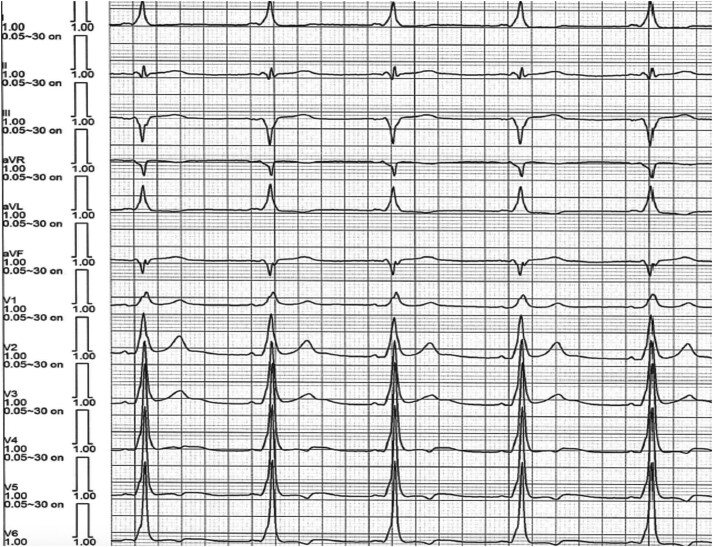
Surface electrocardiogram at the beginning of the second ablation procedure showing pre-excitation with delta wave with superior axis, negative in aVR and positive in aVL, isoelectric in V1 and positive in V2, suggestive of a posteroseptal accessory pathway.

**Figure 5:**
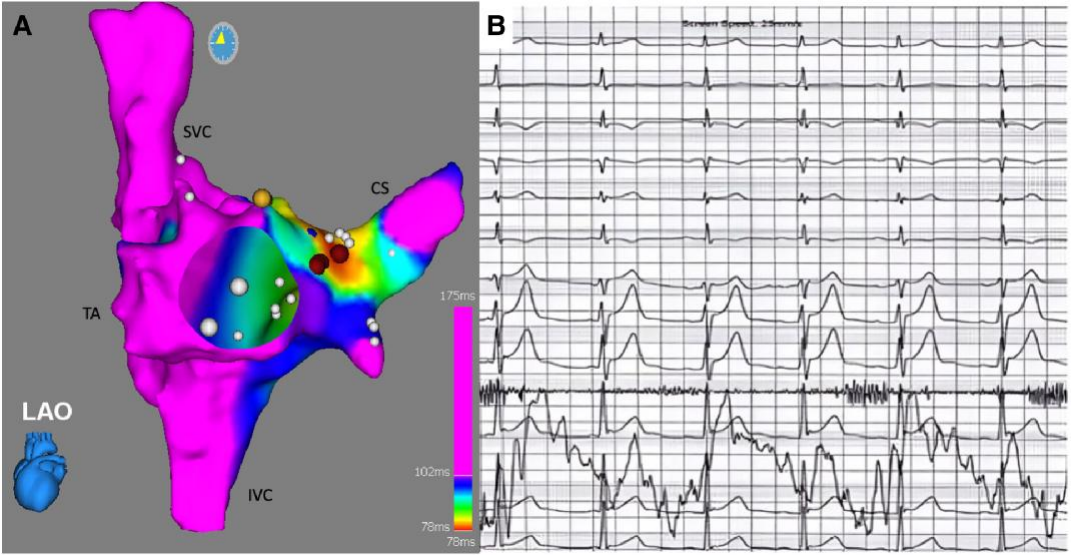
3D electroanatomic image (COLUMBUS™, Microport) showing a continuous atrioventricular activity next to the ostium of the coronary sinus (*A*). Radiofrequency ablation eliminated the conduction through the accessory pathways (*B*). CS, coronary sinus; SVC, superior vena cava; IVC, inferior vena cava; TA, tricuspid annulus.

The patient was discharged the next day, asymptomatic and without evidence of pre-excitation on the ECG. Bisoprolol was suspended and he was not taking any other medication. At 6 months after the ablation, the ECG showed recurrence of ventricular pre-excitation and a new EPS was proposed. The patient was in sinus rhythm with manifest pre-excitation, with negative delta wave in lead aVR, positive in aVL, isoelectric in V1, and positive in V2, suggesting a posteroseptal AP location (*Figure 4*). Apical right ventricular stimulation demonstrated decremental and concentric retrograde conduction, which was exclusively nodal. Incremental right atrial appendage stimulation exacerbated ventricular pre-excitation, with conduction through the AP until an anterograde refractory period of 320 ms. Intracardiac mapping was performed using the COLUMBUS™ system (Microport) (*Figure 5A*). Continuous AV activity was documented at the CS ostium, with a negative monopolar morphology. Radiofrequency was applied using an irrigated FireMagic™ (Microport) catheter (40 W, 15 mL irrigation, maximum temperature of 40°C), eliminating conduction through the AP after four seconds (*Figure 5B*). During a 20-min observation period, there was no recurrence of ventricular pre-excitation. The patient remained asymptomatic with a normal ECG in the 3-month and 1-year follow-ups in our outpatient clinic, without any medication.

## Discussion

The first-line therapy for symptomatic manifest AP is catheter ablation via RF or cryoenergy application. Success rates depend on the AP location, with 90% success for right-sided pathways and up to 97% for left-sided pathways.^5^ However, the literature reports 3–10% of ablations as unsuccessful, with failure to recognize the presence of multiple APs being the most common cause.^3–5^ In our patient, the mechanism explaining the lack of response to the repeated (and higher dose) administration of adenosine could be the use of a second AP as the retrograde limb of the pre-excited AVRT, a rarely described phenomenon known as duodromic AVRT.^6,7^

We believe a duodromic AVRT is the most likely mechanism, as the nodal VA conduction after the first ablation procedure was latent. Unfortunately, the clinical tachycardia was not inducible during the procedure. If the tachycardia had been inducible, a bolus of 30 mg adenosine with AV node block and no alteration or termination of the tachycardia would have confirmed the duodromic mechanism (one AP as the antegrade branch and the other as the retrograde branch). It is also noteworthy that the third AP was not active during the first procedure, even though it was most likely responsible for the clinical tachycardia, as the ECG during tachycardia suggested a right posterior AP. We hypothesize that the third AP's conduction may have been interrupted during the first procedure due to repeated trauma to the CS ostium during catheter positioning. This theory is supported by the observed supraventricular ectopy seen in *Figure 2* and the absence of AP conduction at the end of the first ablation procedure. This fact could also explain why the tachycardia was not inducible during the procedure, since it was likely using the right posterior AP as the anterograde limb. Another explanation for the delayed appearance of this AP near the CS ostium could be the dynamic conduction properties of APs, which may change over time.^8^ While spontaneous loss of pre-excitation is well-documented, the manifestation of a new AP is rare. In our patient, the identification of dual APs during the first EPS was demonstrated by distinct sites of earliest atrial activation, and successful ablation of both APs was achieved.

## Conclusion

Multiple APs present diagnostic and therapeutic challenges. Since they predispose patients to faster atrial and ventricular arrhythmias, their identification and ablation during EPS is crucial, though not always inducible during the procedure. Pre-excited AVRT involving a second AP as the retrograde limb is a rare mechanism known as duodromic AVRT that should be considered when the tachycardia does not respond to adenosine administration.

## Supplementary Material

ytae698_Supplementary_Data

## Data Availability

The data underlying this article are available in the article and in its online Supplementary material.

## Lead author biography

Dr Sofia Jacinto is a Cardiology resident from Lisbon, Portugal. She graduated from NOVA Medical School and has spent the last 5 years working in the Cardiology Department of Santa Marta Hospital, a tertiary university hospital in Lisbon. Currently, Dr Jacinto is embarking on her electrophysiology and cardiac implantable electronic devices (CIED) training in the Arrhythmology Unit. She is also collaborating in the Cardiology remote monitoring clinic, working with pioneering risk stratification algorithms for patients with CIED. Her record also encompasses scientific presentations at national and international congresses and co-investigator roles in clinical trials.

## Notes

**Consent:** The authors confirm that the written consent for submission and publication of this case report, including images and associated text, has been obtained from the patient in line with COPE guidance.

**Funding:** The authors have not declared a specific grant for this research from any funding agency in the public commercial or not-for-profit sectors.
