# Supplementary figures and images for: Duodromic atrioventricular reentry tachycardia: a case report of a rare adenosine insensitive supraventricular tachycardia
Source: Eur Heart J Case Rep. 2024 Dec 30;9(1):ytae698. doi: 10.1093/ehjcr/ytae698 (PMC11718398; doi:10.1093/ehjcr/ytae698)

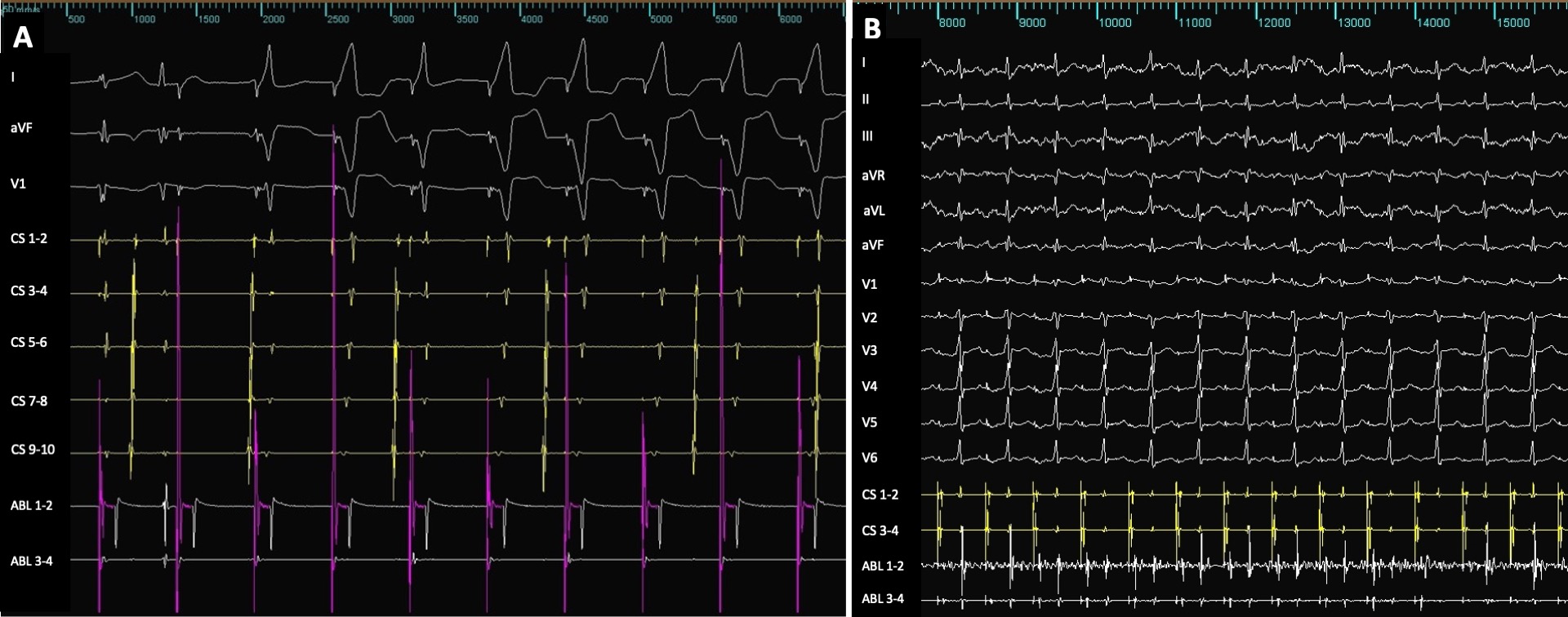

Supplement: ytae698_Supplementary_Data [file ytae698_supplementary_data.zip › Suppl1.jpg]

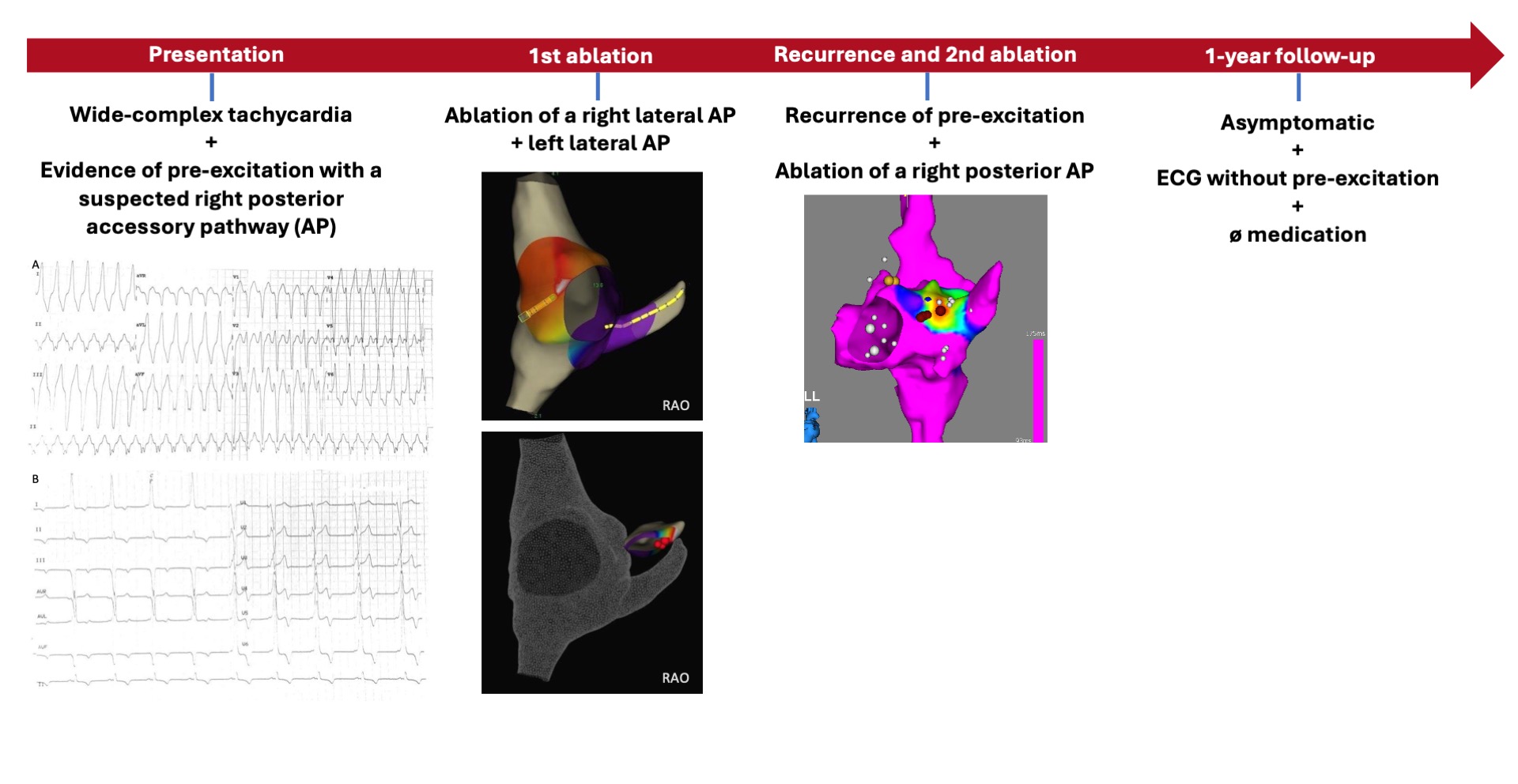

Supplement: ytae698_Supplementary_Data [file ytae698_supplementary_data.zip › Suppl2.jpg]
